# Utility of Adrenal Vein Sampling With and Without Ultra‐Low Dose ACTH Infusion in the Diagnostic Evaluation of Primary Aldosteronism

**DOI:** 10.1002/edm2.70001

**Published:** 2024-08-29

**Authors:** Christopher A. Preston, Eric X. Z. Yong, Benjamin Marginson, Stephen G. Farrell, Matthew P. Sawyer, Hikaru Hashimura, Maresa M. Derbyshire, Richard J. MacIsaac, Nirupa Sachithanandan

**Affiliations:** ^1^ Department of Endocrinology & Diabetes St Vincent's Hospital Melbourne Fitzroy Victoria Australia; ^2^ Department of Medicine The University of Melbourne St Albans Victoria Australia; ^3^ Department of Radiology St Vincent's Hospital Melbourne Fitzroy Victoria Australia; ^4^ Department of Radiology Peter MacCallum Cancer Centre Parkville Victoria Australia; ^5^ Department of Surgery St Vincent's Hospital Melbourne Fitzroy Victoria Australia; ^6^ Department of Medicine The University of Melbourne Fitzroy Victoria Australia

**Keywords:** ACTH, ACTH AVS, adrenal vein sampling, cortisol, primary aldosteronism, ultra‐low dose cosyntropin

## Abstract

**Background:**

Adrenal vein sampling (AVS), integral to identifying surgically remediable unilateral primary aldosteronism (PA), is technically challenging and subject to fluctuations in cortisol and aldosterone secretion. Intra‐procedural adrenocorticotropic hormone (ACTH), conventionally administered as a 250‐μg bolus and/or 50 μg per hour infusion, increases cortisol and aldosterone secretion and can improve AVS success, but may cause discordant lateralisation compared to unstimulated AVS.

**Aims:**

To assess if AVS performed with ultra‐low dose ACTH infusion causes discordant lateralisation.

**Methods:**

Here, we describe our preliminary experience using an ultra‐low dose ACTH infusion AVS protocol. We retrospectively reviewed the results of consecutive AVS procedures (*n* = 37) performed with and without ultra‐low dose ACTH (1‐μg bolus followed by 1.25 μg per hour infusion).

**Results:**

Bilateral AV cannulation was successful in 70% of procedures pre‐ACTH and 89% post‐ACTH (*p* < 0.01). Sixty‐nine percent of studies lateralised pre‐ACTH and 55% post‐ACTH, improving to 79% when both groups were combined. Lateralisation was discordant in 11 cases, including eight in which lateralisation was present only on basal sampling, and three in which lateralisation occurred only with ACTH stimulation.

**Discussion:**

Overall, the decrease in lateralisation rates with ACTH was higher than previously reported for some protocols utilising conventional doses of ACTH. Our results suggest that AVS performed with ultra‐low dose ACTH can cause discordant lateralisation similar to AVS performed with conventional doses of ACTH.

**Conclusion:**

Prospective studies directly comparing low and conventional dose ACTH AVS protocols and long‐term patient outcomes are needed to help define the optimal ACTH dose for accurate PA subtyping.

## Graphical abstract

- AVS performed with ultra‐low dose ACTH may mask lateralisation and does not obviate the need for non‐ACTH AVS.
- Combined AVS with and without ultra‐low dose ACTH can improve the diagnostic yield of the procedure, identifying additional cases of surgically remediable unilateral PA (*Lancet Diabetes Endocrinol*. 2015, 3, 296).

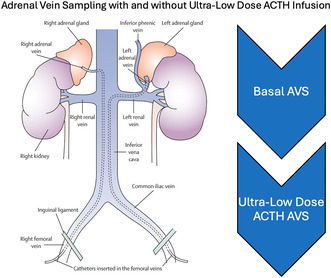

**Summary**

**What Is Known About This Topic**

- AVS is technically challenging and subject to fluctuations in cortisol and aldosterone secretion.
- Intra‐procedural ACTH at conventional doses increases cortisol and aldosterone secretion and can improve AVS success, however, may also mask lateralisation of unilateral PA.

**What This Study Adds**

- AVS performed with ultra‐low dose ACTH may mask lateralisation and does not obviate the need for non‐ACTH AVS.
- Combined AVS with and without ultra‐low dose ACTH can improve the diagnostic yield of the procedure, identifying additional cases of surgically remediable unilateral PA.

## 1 Introduction

Primary aldosteronism (PA) is the most common cause of secondary hypertension, accounting for at least 10% of cases of hypertension in the general population [1, 2] and up to 30% of refractory cases [3, 4] and is associated with increased cardiovascular morbidity and mortality compared with primary hypertension of similar severity [5, 6].

Whereas PA caused by bilateral hyperaldosteronism is best treated medically, that caused by an aldosterone‐producing adenoma or hyperplasia of a single adrenal gland is surgically curable by unilateral adrenalectomy, which also relieves the associated excess cardiovascular risk [7, 8, 9, 10]. Therefore, accurate diagnosis and subtyping of PA is essential to guide optimised and targeted treatments and minimise complications. However, distinguishing unilateral from bilateral disease remains expensive and technically challenging.

Cross‐sectional imaging via computed tomography (CT) has low sensitivity and specificity for subtyping PA as either unilateral or bilateral [11, 12]. Hence, adrenal vein sampling (AVS), which provides a direct comparison of aldosterone concentration from each adrenal vein (AV) corrected for cortisol concentration, is integral to identifying cases of surgically remediable unilateral PA [13, 14, 15]. However, cannulation of the right AV can be technically challenging, limiting the overall diagnostic success of AVS procedures [16].

In many centres, AVS is performed with the administration of synthetic adrenocorticotropic hormone (ACTH, cosyntropin), conventionally as a 250 μg bolus or 50 μg per hour infusion for the duration of the procedure, or with a combination of bolus followed by continuous infusion [17, 18, 19, 20, 21]. However, a consensus on optimal ACTH dosing is currently lacking, with protocols differing significantly between centres [22, 23, 24]. The advantages of using ACTH during AVS include maximising adrenal cortisol secretion and the consequent adrenal to peripheral cortisol gradient, thereby improving the recognition of successful AV sampling [17, 18, 19, 20, 21], stimulation of aldosterone secretion in unilateral PA to maximise the aldosterone gradient and thus avoid the risk of sampling during a relatively quiescent phase of aldosterone secretion, and minimising intra‐procedural stress‐induced fluctuations in cortisol and aldosterone secretion, especially during sequential adrenal vein cannulation [20, 21, 22]. The drawback of ACTH, however, is that it may simultaneously mask lateralisation of unilateral PA, possibly due to stimulation of aldosterone secretion from the contralateral adrenal [25, 26], resulting in the false subtyping of unilateral PA as bilateral, thereby precluding surgical cure. In addition, ACTH may also cause discordant lateralisation in asymmetrical bilateral PA, the long‐term impact of which remains unclear [27]. Consistent with this, published series from centres that perform AVS both before and after the administration of ACTH have consistently shown discordant lateralisation results in up to one quarter of patients (mostly unilateral PA pre‐ACTH to bilateral PA post‐ACTH, but rarely bilateral PA to unilateral or side reversal from right to left or vice versa) [28, 29], with ACTH stimulation equally likely to augment, diminish or preserve the aldosterone gradient, even in patients who consistently lateralised excess aldosterone secretion to one adrenal gland [27, 29].

The majority of studies addressing the issue of discordant lateralisation between pre‐ and post‐ACTH sampling have, however, been performed utilising conventional and supraphysiological doses of ACTH only. It is plausible that a much lower dose of ACTH closer to physiological levels may result in lower rates of discordant lateralisation without affecting procedural success. However, it remains unknown if AVS performed with ultra‐low dose ACTH will have the same effect on lateralisation. Here we describe our AVS experience using an ultra‐low dose ACTH infusion protocol (1.25 μg per hour infusion following a 1 μg bolus to ensure adequate priming of adrenal aldosterone secretion).

The primary aim of our study was to assess if AVS performed with ultra‐low dose ACTH infusion causes discordant lateralisation after a change in protocol to include unstimulated AVS to stimulated AVS at our institution. Secondary aims included assessing the overall utility of AVS performed both with and without ultra‐low dose ACTH in terms of correlation with surgical outcomes, as well as evaluating the impact of adding basal AVS to ultra‐low dose ACTH‐stimulated AVS in terms of procedure duration, bilateral AV cannulation success and overall lateralisation rates.

## 2 Methods

We conducted a retrospective review of the medical records of patients with biochemically proven PA according to current Endocrine Society guidelines [17] that underwent consecutive AVS procedures at our institution (St Vincent's Hospital Melbourne, a tertiary referral centre and University teaching hospital) both with and without ultra‐low dose ACTH infusion between October 2020 and December 2022 (*n* = 37).

Patients were identified from a departmental database for inclusion in the study. Between October 2020 and December 2020, according to our institution's protocol, all patients referred for AVS who were under 40 years of age, or under 50 years of age with hypokalaemia or an adrenal lesion on CT, underwent AVS both with and without ACTH infusion (*n* = 3). From 2021 onwards, this protocol was amended such that all patients below age 60 referred for AVS, or those above age 60 with a lesion on adrenal CT or hypokalaemia, underwent both unstimulated and stimulated procedures (*n* = 34). All others underwent ACTH‐stimulated AVS only as described previously [30].

Data including patient demographics, history of hypertension or hypokalaemia, baseline potassium level, number and type of antihypertensive medications, potassium supplements, biochemical confirmation of PA, adrenal imaging findings, and all AVS data, including bilateral AV cannulation success, lateralisation results and overall procedure duration, were extracted from medical records. We then compared the results of AVS performed without ACTH with the results of AVS performed under the stimulation of ultra‐low dose ACTH infusion in the same patient. For the outcome of procedure duration, a matching number of patients who underwent AVS according to our previous protocol (AVS was performed with ACTH infusion only during this period) were also identified from the 12‐month period immediately prior to the current study, for inclusion as historical controls.

### 2.1 AVS Protocol

The AVS protocol at our institution is adapted from that of Espiner et al. [31] with modifications, with all procedures performed by one of two specialist interventional radiologists, each with extensive AVS experience and a focused expertise in the technique.

Interfering medications were ceased for several weeks prior to AVS in accordance with current recommendations [17], and potassium supplemented in 28 of 37 patients as required to maintain normokalaemia (Table S1). All patients also underwent contrast‐enhanced, fine‐slice (1 mm thickness), portal venous phase CT of the adrenal glands prior to AVS, to facilitate AV localisation and assessment of adrenal gland morphology, and a baseline ACTH measurement on the day of the procedure, immediately prior to the study.

Ultra‐low dose ACTH infusion was prepared as described in Figure S1 and AVS was performed between 9 AM and 2 PM, depending on availability of resources. Basal AVS without ACTH stimulation was performed initially, with catheter placement confirmed utilising intra‐procedural quick cortisol assay (QCA, AVS Accuracy Kit, Trust Medical Co., Kyoto, Japan) following internal validation (M. P. Sawyer, C. A. Preston, E. X. Z. Yong, B. Marginson, S. G. Farrell, M. M. Derbyshire, R. J. MacIsaac, & N. Sachithanandan, unpublished data). Aldosterone and cortisol samples were then collected sequentially from the left and then the right adrenal vein, coupled with simultaneous peripheral vein samples. This sampling process was then repeated under ACTH stimulation, administered as a 1 μg intravenous bolus followed by an infusion at 1.25 μg per hour, with sampling resuming 20 min following the bolus (Figure S1). During the infusion, the catheter tips were withdrawn and placed near the orifice of the adrenal veins without completely engaging the vein to prevent thrombosis. If catheter placement was not confirmed during basal sampling, samples were considered non‐diagnostic, and further attempts at cannulation were continued until successful or ACTH‐stimulated sampling was commenced at the discretion of the interventional radiologist.

At the completion of the study blood samples were sent for analysis of cortisol and aldosterone concentrations.

### 2.2 Laboratory Measurements

Serum potassium concentration was measured by indirect ion selective electrodes using the Beckman Coulter AU5800 Analyzer, plasma aldosterone and renin concentrations by automated chemiluminescence using the DiaSorin Liaison XL immunoassay (CLIA, Salugia, Italy) and cortisol concentration by the Abbott Architect chemiluminescent microparticle immunoassay (Abbott Laboratories, Illinois, USA). Adrenocorticotropic was measured using the Siemens Immulite immunoassay (Siemens Healthineers, Erlangen, Germany).

### 2.3 AVS Interpretation

Successful AV cannulation (the selectivity index) was defined by an AV to peripheral vein cortisol ratio ≥2.0 pre‐ACTH and ≥4.0 post‐ACTH, demonstrating at least two‐ or four‐fold increases in cortisol in the adrenal veins compared to peripheral veins, respectively. Lateralisation was defined by an aldosterone‐to‐cortisol ratio of the dominant to non‐dominant adrenal vein ≥3.0 pre‐ACTH and ≥4.0 post‐ACTH (the lateralisation index), indicating unilateral aldosterone excess, while a ratio of <2.0 pre‐ or <3.0 post‐ACTH was considered indicative of bilateral aldosterone excess. Lateralisation ratios between 2.0 and 3.0 pre‐ or 3.0 and 4.0 post‐ACTH were considered equivocal. Contralateral suppression was defined by an aldosterone‐to‐cortisol ratio of the non‐dominant adrenal vein to the peripheral veins of ≤1.0 (Table S2).

### 2.4 Follow‐Up Data

Surgical pathology following unilateral adrenalectomy was confirmed via histology reports, with follow‐up biochemistry including serum potassium and aldosterone and renin ratio measured at 3–6 months post‐operatively, and clinical data regarding resolution or persistence of hypertension, and requirement for ongoing antihypertensives or potassium supplementation reported where available.

### 2.5 Statistical Analysis

Categorical data are reported as number (%), and continuous data as mean ± standard deviation if normally distributed, otherwise as median and interquartile range (IQR). Statistical analysis was performed using Microsoft Excel (version 16, Microsoft Corporation) or STATA (version 16.1 STATA LP College Station), with between group differences compared by two‐sample Student's *t* test, Mann–Whitney *U* test or chi‐square test as appropriate. *p* < 0.05 were considered statistically significant.

## 3 Results

A total of 37 AVS procedures using both ACTH and non‐ACTH protocols were performed during the study period and included in the study.

Baseline characteristics of the study participants are presented in Table 1. Mean age of participants was 47.6 years and there was a preponderance of females (62%). All participants had a history of hypertension, with 57% also having a history of hypokalaemia, and 59% had an adrenal abnormality demonstrated on CT (43% had both hypokalaemia and an imaged adrenal abnormality). Median ACTH levels at baseline prior to commencing the procedure was 15 ng/L (range 6–26 ng/L). Adrenal vein sampling was performed in the morning in 20 patients and in the afternoon in 17.

**TABLE 1:** Baseline subject characteristics.

Baseline subject characteristics		
Patients (*n*)		37
Age (years)		47.6 ± 9.2
Female (%)		23 (62%)
SBP		148 ± 14
DBP		92 ± 11
History of hypertension (%)		37 (100%)
Number of antihypertensives		1.9 ± 0.8
History of hypokalaemia (%)		21 (57%)
Serum K (mmol/L)		3.38 ± 0.45
Renin (mIU/L)	(RR 4.4–46.0)	2.6 (1.8, 4.9)
Aldosterone (pmol/L)	(RR 61–978)	729 (526, 950)
ARR (pmol/mIU)	(RR <70)	232 (131, 406)
Adrenal abnormality on CT (%)^a^		22 (59%)

*Note:* Values are mean ± standard deviation or median (interquartile range).

Abbreviations: ARR, aldosterone‐to‐renin ratio; CT, computed tomography; DBP diastolic blood pressure; K, potassium; RR, reference range; SBP, systolic blood pressure.

^a^ Adrenal abnormality included gland asymmetry or hyperplasia, unilateral adrenal nodule or adenoma.

**FIGURE 1:**
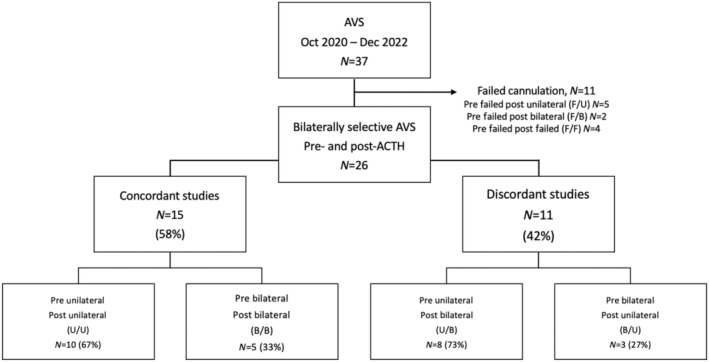
Study flow chart and PA subtyping results. AVS, adrenal vein sampling; B/B, Bilateral to bilateral; B/U, Bilateral to unilateral; F/B, Failed to bilateral; F/U, Failed to unilateral; post, post‐ACTH sampling; pre, pre‐ACTH sampling; U/B, Unilateral to bilateral; U/U, Unilateral to unilateral.

Adrenocorticotropic markedly stimulated cortisol and aldosterone secretion from both adrenals, as evidenced by an increase in cortisol and aldosterone levels in the adrenal and peripheral veins compared to baseline (Table S3). Bilateral AV cannulation was successful in 70% (*n* = 26) of procedures pre‐ACTH and 89% (*n* = 33) of procedures post‐ACTH (Table 2, *p* < 0.01). Of the 11 procedures with unsuccessful cannulations during unstimulated AVS, seven were unsuccessful only during unstimulated sampling (three failed left AV, four failed bilaterally), with bilateral cannulation subsequently achieved under ACTH stimulation, while the remaining four studies were consistently unsuccessful during both unstimulated and stimulated sampling (cannulation failure rates: two failed right AV pre and post‐ACTH, one failed bilaterally pre‐ACTH and right AV post‐ACTH, one failed left AV pre and post‐ACTH, Figure 1, Table S4).

**TABLE 2:** Rates of bilaterally successful AVS pre‐ and post‐ACTH and proportion of studies lateralised.

	Pre‐ACTH	Post‐ACTH	Combined	*p* pre‐ACTH vs. post‐ACTH	*p* pre‐ACTH vs. combined	*p* post‐ACTH vs. combined
Successful cannulation	26/37 (70%)	33/37 (89%)	33/37 (89%)	**<0.01**	**<0.01**	*p* = ns
Lateralised	18/26 (69%)	18/33 (55%)	26/33 (79%)	0.07	0.11	**<0.01**

*Note:* *p* < 0.05 were considered statistically significant (bold).

Overall, 69% of bilaterally successful studies fulfilled lateralisation criteria during unstimulated sampling and 55% met criteria for lateralisation following stimulation (*p* = 0.07). When both groups were combined, the total number of lateralised procedures increased to 79% of studies overall (Table 2, *p* < 0.01 combined vs. post‐ACTH, *p* = 0.11 combined vs. pre‐ACTH).

Of the 26 patients in whom AVS was bilaterally successful during both unstimulated and stimulated sampling, lateralisation was possible in 18 (70%) pre‐ACTH and in 13 (50%) post‐ACTH infusion (Figure 1). Lateralisation was concordant with and without ACTH stimulation in 15 (58%) of cases, including 10 (67%) which consistently lateralised to one adrenal, and five (33%) which consistently remained non‐lateralised, demonstrating bilateral hyperaldosteronism (Figure 1, Table 3). Of the 10 cases which consistently lateralised, all were subsequently confirmed histologically as adrenal adenomas, with eight (80%) demonstrating normalisation of aldosterone‐to‐renin ratios and all achieving normokalaemia following unilateral adrenalectomy (Tables 3 and 4).

**TABLE 3:** Surgical pathology results and post‐operative outcomes based on lateralisation results in patients with bilaterally selective AVS (*n* = 26) before and after ACTH stimulation.

AVS o/c	*n*	Surgery	Histology			K			ARR		
			Adenoma	Hyperplasia	Other^a^	Pre	Post	*p*	Pre	Post	*p*
U/U	10	10	10	0	0	3.11 ± 0.45	4.44 ± 0.35	**<0.01**	695 ± 1020	24 ± 27	**0.04**
U/B	8	5	3^b^	1	1	3.75 ± 0.31	4.26 ± 0.34	**<0.01**	262 ± 259	47 ± 40	**0.02**
B/U	3	3	3	0	0	3.27 ± 0.55	4.66 ± 0.15	0.06	252 ± 117	15 ± 7	0.08
B/B	5	0	N/A	N/A	N/A	3.40 ± 0.31	N/A	N/A	212 ± 98	N/A	N/A
Total	26	18/26	16/18	1/18	1/18	3.38 ± 0.47	4.43 ± 0.34	**<0.01**	418 ± 668	28 ± 29	**<0.01**

*Note:* *p* < 0.05 were considered statistically significant (bold).

Abbreviations: ARR, aldosterone‐to‐renin ratio (pmol/mIU); AVS o/c, adrenal vein sampling outcome; B/B, Bilateral to bilateral; B/U, Bilateral to unilateral; K, potassium (mmol/L); N/A, not applicable; post, post‐adrenalectomy; pre, pre‐adrenalectomy; U/B, Unilateral to bilateral; U/U, Unilateral to unilateral.

^a^ Adrenocortical nodular disease (ACND).

^b^ Adenoma and aldosterone‐producing micronodules, both staining positive for Cyp11B2 (aldosterone synthase).

**TABLE 4:** Concordant lateralised studies and post‐operative data in patients proceeding to surgery.

Pt	Basal AVS		ACTH AVS		Outcomes				
	Lateralisation	CLS	Lateralisation	CLS	Surgery	Histology	Baseline K	Post‐op K	Post‐op ARR
1	Lateralised	Y	Lateralised	Y	Yes	Adenoma	Normal	Normal	Normal
2	Lateralised	Y	Lateralised	Y	Yes	Adenoma	Low	Normal	Normal
3	Lateralised	Y	Lateralised	Y	Yes	Adenoma	Low	Normal	Normal
4	Lateralised	Y	Lateralised	Y	Yes	Adenoma	Normal	Normal	Normal
5	Lateralised	Y	Lateralised	Y	Yes	Adenoma	Low	Normal	Normal
6	Lateralised	Y	Lateralised	Y	Yes	Adenoma	Low	Normal	Normal
7	Lateralised	Y	Lateralised	Y	Yes	Adenoma	Low	Normal	High
8	Lateralised	Y	Lateralised	Y	Yes	Adenoma	Low	Normal	Normal
9	Lateralised	N	Lateralised	Y	Yes	Adenoma	Low	Normal	Normal
10	Lateralised	Y	Lateralised	Y	Yes	Adenoma	Low	Normal	Unknown

Abbreviations: ARR, aldosterone‐to‐renin ratio; AVS, adrenal vein sampling; CLS, contralateral suppression; K, potassium; N, no; Pt, patient; Y, yes.

Lateralisation results were discordant in 11 cases (42%), including eight (73%) in which lateralisation was present during unstimulated sampling but not during subsequent stimulation by ACTH (unilateral (U) to bilateral (B) group), and three (27%) in which lateralisation was identified only with ACTH stimulation (bilateral (B) to unilateral (U) group, Table 3). Of the eight cases in which lateralisation was present only during basal sampling (U to B group), five proceeded to surgery, with two having an adrenocortical adenoma confirmed histologically, one having an adenoma and aldosterone‐producing micronodules (both staining positive for Cyp11B2, aldosterone synthase) and another adrenal hyperplasia, and one patient undergoing surgery to the non‐lateralising contralateral gland for suspected mild autonomous cortisol secretion (MACS) arising from the contralateral side. The three remaining patients in this group have elected for medical management (Table 5). The three patients who had bilateral PA on unstimulated sampling but lateralised on ACTH stimulation (B to U group) all had adrenocortical adenomas confirmed at surgery and had normalisation of ARR and hypokalaemia following surgery. Of the seven studies in which cannulation was bilaterally successful only post‐stimulation, lateralisation was identified in five cases, all of whom proceeded to surgery. Of these, four were subsequently confirmed to have adenomas on histology with resolution of hypokalaemia post‐operatively. The fifth patient had hyperplasia on histology and continues to have an elevated ARR post‐operatively (Table 6).

**TABLE 5:** Discordant studies and post‐operative outcomes in patients undergoing unilateral adrenalectomy.

Pt	Basal AVS		ACTH AVS		Outcomes				
	Lateralisation	CLS	Lateralisation	CLS	Surgery	Histology	Baseline K	Post‐op K	Post‐op ARR
Discordant—Lateralisation Masked by ACTH									
1	Lateralised	Y	Non‐lateralised	N/A	Yes	Hyperplasia	Normal	Normal	Normal
2	Lateralised	Y	Non‐lateralised	N/A	Yes	Adenoma	Low	Normal	Normal
3	Lateralised	Y	Non‐lateralised	N/A	Yes	Adenoma	Normal	Normal	Normal
4	Lateralised	Y	Non‐lateralised	N/A	No	N/A	Normal	N/A	N/A
5	Lateralised	N	Non‐lateralised	N/A	Yes	Adenoma^a^	Low	Normal	High^b^
6	Lateralised	Y	Non‐lateralised	N/A	No	N/A	Normal	N/A	N/A
7	Lateralised	N	Non‐lateralised	N/A	Yes^c^	ACND	Normal	Normal	U/A
8	Lateralised	N	Non‐lateralised	N/A	No	N/A	Normal	N/A	N/A
Discordant—Lateralised only with ACTH									
1	Non‐lateralised	N/A	Lateralised	Y	Yes	Adenoma	Low	Normal	Normal
2	Non‐lateralised	N/A	Lateralised	Y	Yes	Adenoma	Low	Normal	Normal
3	Non‐lateralised	N/A	Lateralised	Y	Yes	Adenoma	Normal	Normal	Normal

Abbreviations: ACND, adrenocortical nodular disease; ARR, aldosterone‐to‐renin ratio; AVS, adrenal vein sampling; CLS, contralateral suppression; K, potassium; N, no; N/A, not applicable; Pt, patient; U/A, unavailable; Y, yes.

^a^ Adenoma and aldosterone‐producing micronodules, both staining positive for Cyp11B2 (aldosterone synthase).

^b^ ARR improved but remained elevated post‐surgery.

^c^ Underwent contralateral subtotal adrenalectomy for suspected mild autonomous cortisol secretion (MACS), PA treated with mineralocorticoid antagonist.

**TABLE 6:** Outcomes of unsuccessful AVS studies.

Failed cannulation during unstimulated and stimulated sampling						
Pt	AVS result	Surgery	Histology	Baseline K	Post‐op K	Post‐op ARR
1	Failed	Yes	Adenoma	Normal	Normal	Normal
2	Failed	No	N/A	Low	N/A	N/A
3	Failed	No	N/A	Low	N/A	N/A
4	Failed	No	N/A	Normal	N/A	N/A

Successful cannulation during stimulated sampling only							
Pt	AVS result	CLS	Surgery	Histology	Baseline K	Post‐op K	Post‐op ARR
1	Non‐Lateralised	N/A	No	N/A	Low	N/A	N/A
2	Non‐Lateralised	N/A	No	N/A	Normal	N/A	N/A
3	Lateralised	Y	Yes	Hyperplasia	Normal	Normal	High
4	Lateralised	Y	Yes	Adenoma	Low	Normal	Unknown
5	Lateralised	Y	Yes	Adenoma	Low	Normal	Unknown
6	Lateralised	Y	Yes	Adenoma	Low	Normal	Unknown
7	Lateralised	Y	Yes	Adenoma	Low	Normal	Unknown

Abbreviations: ARR, aldosterone‐to‐renin ratio; AVS, adrenal vein sampling; CLS, contralateral suppression; K, potassium; N, no; N/A, not applicable; Pt, patient; Y, yes.

Procedural time was increased by an average of 45 min in the combined stimulated and unstimulated sampling protocol compared to the ACTH only protocol (143 ± 33 min vs. 98 ± 36 min, Table S5, *p* < 0.01) and did not differ between proceduralists (Table S6), or between the first 10 and last 27 procedures (Table S7).

Overall, radiologist A performed 70% of all AVS procedures. Cannulation success rates were similar between interventionalists, with improvements with ACTH in both (Table S8).

## 4 Discussion

Adrenal vein sampling, the gold standard procedure for identifying surgically curable cases of unilateral PA, is technically challenging, and the ideal protocol for optimising its diagnostic performance remains uncertain.

While the addition of intra‐procedural ACTH has been shown to improve successful AV sampling by maximising adrenal cortisol secretion and the consequent adrenal to peripheral cortisol gradient which can be low during the basal sampling, it can lead to discordant lateralisation. In some cases, this may mask lateralisation of unilateral PA, resulting in the false subtyping of unilateral PA as bilateral [27, 28, 29]. Less commonly, side reversal of lateralised PA as well as conversion of bilateral PA to unilateral PA have also been reported [28, 29].

Here, we report our real‐world experience of implementing a new AVS protocol adding basal AVS to ultra‐low dose ACTH‐stimulated AVS (1 μg bolus followed by infusion at 1.25 μg per hour with sampling commencing 20 min post bolus) to improve the diagnostic success of the procedure. This is a modification on that published by Espiner et al., which showed ACTH infusion at 1.25 μg per hour commencing 45 min before sampling robustly increased selectivity [30, 31]. The modified dosing we adopted in this study consistently increased selectivity in at least one adrenal vein in all AVS studies with no patient having bilateral selectivity index <4 on post‐ACTH sampling indicating that failed cannulations in the studies that were unsuccessful post‐ACTH were due to technical reasons alone rather than due to a lack of stimulation of cortisol secretion by the low dose ACTH administered during the study. In addition, aldosterone levels in the adrenal vein also increased by a median 12‐fold (IQR 7‐ to 20‐fold) in post‐stimulation samples compared with levels obtained at baseline.

Without ACTH stimulation, the success rate for bilateral cannulation in our study was 70%, similar to the rates reported in unstimulated sampling in other studies [23, 28]. This improved to 89% following stimulation with ultra‐low dose ACTH, which is comparable to the rates reported with ACTH stimulation at much higher conventional doses in high volume centres [23, 28]. Moreover, in our series, AVS was bilaterally successful only following ACTH stimulation in seven cases with five of the seven cases having a selectivity index >1.64 on basal AVS (Table S4), indicating that ACTH stimulation is invaluable in a subset of patients where basal sampling is deemed unsuccessful due to high basal IVC cortisol levels and a low adrenal to peripheral cortisol gradient. The resultant low selectivity index is due to a high denominator in the selectivity ratios, despite the catheters being positioned correctly within the adrenal vein. Post‐stimulation, adrenal cortisol levels are inflated several‐fold above the peripheral venous cortisol making these studies diagnostically useful and impacting patient management.

Reassuringly, all cases in which lateralisation was consistently demonstrated in both unstimulated and stimulated protocols were subsequently confirmed histologically as adrenocortical adenomas, with the majority (80%) having biochemical cure (normalisation of ARR) and all having resolution of hypokalaemia following adrenalectomy. Taken together, these data suggest an excellent level of confidence in this combination of concordant results predicting surgically remediable disease. Furthermore, there were eight cases in which lateralisation was identified only with ACTH stimulation (three who lateralised on post‐ACTH sampling only and five in whom AVS was bilaterally successful only with ACTH), consistent with previous studies demonstrating improved lateralisation with ACTH in a subset of patients with unilateral PA [26, 28]. Overall, our lateralisation rates were also comparable to those reported in the literature, and our available surgical outcomes, although modest in number and incomplete, were also consistent with those achieved following AVS with ACTH stimulation at conventional doses [28, 29].

Conversely, however, there were also eight cases with discordant lateralisation, in which lateralisation was identified in basal AVS, but not with ACTH stimulation, as has been reported with conventional doses [23, 27, 28, 29]. However, all cases in this subgroup that proceeded to adrenalectomy were subsequently confirmed to have either adrenocortical adenomas or hyperplasia histologically, with normalisation of aldosterone and renin levels in most (75%) and normokalaemia in all post‐operatively, consistent with biochemical cure or benefit following adrenalectomy. Taken together, our data adds to the existing body of literature suggesting that ACTH even if administered as ultra‐low dose infusion may mask lateralisation in some cases, however, also identifies additional cases of surgically remediable unilateral PA by improving selectivity, which is a clinically significant outcome. In our study we used a lateralisation index cut‐off of ≥3.0 for unstimulated sampling whereas many centres use a lower cut‐off of ≥2.0 to diagnose lateralised PA [23, 27]. However, our cut‐off of ≥3.0 is not dissimilar to many centres using similar parameters and demonstrating excellent post‐operative outcomes [23, 29, 32]. Adjusting the pre‐stimulation lateralisation index to ≥2.0 would increase the proportion of lateralised PA on basal sampling to 88%, and discordance to 54% (1 of 3 B/U would become U/U, and 4 of 5 B/B would become U/B).

Notably, our stimulated and unstimulated lateralisation results were discordant in 42% of cases, which was slightly higher than the rates of up to 30% reported following stimulation with conventional doses of ACTH [25, 26, 28, 29]. Conceivably, this may reflect potential differences in study populations, a small sample size, local AVS protocols, underlying molecular pathogenesis of aldosterone‐producing adenomas and heterogeneity of aldosterone secretory response of aldosterone‐producing adenomas to varying doses of ACTH [29]. Future studies which include routine testing for known aldosterone‐driver mutations and expression of MCR2 may be informative.

Any analysis of dual sampling pre‐ and post‐ACTH should include a cost–benefit analysis. The cost of ACTH is AUD 308.00 per 1 mg/mL vial in Australia, and protocols which include high dose bolus or continuous infusion may incur a higher cost due to more usage. Our protocol's use of approximately 1% of conventional doses of ACTH per diagnostic procedure, may prove useful in improving the overall health care economics of this widely utilised diagnostic procedure, especially if cheaper ACTH vials with lower dosage amounts become commercially available and may result in a cost saving of up to 5%–10% per procedure.

Our study has several strengths. It is the first to our knowledge to successfully compare AVS with a new ultra‐low dose ACTH infusion protocol to basal AVS performed in the same patient and provides valuable real‐world data demonstrating the utility of ultra‐low dose ACTH in AVS, when combined with basal AVS. Additionally, despite the limited number of patients who proceeded to surgery, our study is the first to our knowledge to compare basal and ultra‐low dose ACTH‐stimulated lateralisation rates for surgical outcomes. Patients with concordant lateralisation on both unstimulated and stimulated AVS accounted for just over half of all patients who proceeded to surgery, whilst three other patients who lateralised only with ultra‐low dose ACTH also had adrenal adenomas confirmed on histology with normalisation of aldosterone‐to‐renin ratios on post‐operative biochemistry. All five patients who lateralised with ACTH only following failed cannulation during unstimulated sampling also proceeded to surgery, with four confirmed as adrenal adenomas and one as hyperplasia, with four of whom also having resolution of hypokalaemia post‐operatively. The remainder of the operated patients included five patients who lateralised on unstimulated but not stimulated AVS, three of whom achieved normalisation of aldosterone and renin levels post‐operatively (aldosterone‐to‐renin ratio (ARR) improved by 50% but remained elevated in the fourth), with the fifth undergoing surgery to the contralateral gland for suspected MACS. All patients in this subgroup who were hypokalaemic pre‐operatively became normokalaemic post‐surgery. Notably, in centres where only basal or ACTH‐stimulated results are considered, but not both, patients falling into either of these discordant lateralisation subgroups would have missed the opportunity for surgical benefit.

Limitations of our findings include the retrospective, non‐randomised and single‐centre design of our study, selection of patients who were most likely to have unilateral disease on demographics and clinical parameters (age, CT findings, hypokalaemia), small sample size and the limited number of patients with discordant results proceeding to surgery. Many of our patients were referred to our centre for AVS only by external endocrinologists and had surgery at external centres leading to heterogeneity in post‐operative follow‐up. Therefore, most operated patients lack clinical (blood pressure) outcome measures based on PASO criteria [10]. Cyp11B2 immunohistochemistry which would be informative was not routinely performed on resected samples at our institution during the study period [33]. We also used historical controls for the outcome of procedure duration. Our patients were not routinely screened for pre‐existing MACS although no patient in our series had a suppressed ACTH level at baseline (<5 ng/dL) indicating that if MACS was present, it was likely to be mild and insufficient to suppress adrenal cortisol secretion substantially to affect selectivity or lateralisation ratios during basal AVS [34] or ACTH‐stimulated AVS [35] and, therefore, unlikely to cause misclassification. Like all other AVS studies, our findings are also limited by the fact that patients considered to have bilateral disease by AVS do not proceed to surgery, thus histopathological confirmation is not available to correlate with AVS results for this subgroup.

In conclusion, our preliminary study has shown that ultra‐low dose ACTH stimulation causes discordant lateralisation similar to AVS performed with standard dose ACTH. However, combined AVS with and without ultra‐low dose ACTH improved the overall diagnostic yield of the procedure in the real‐world setting in a select cohort of patients with a high likelihood of unilateral disease, with an acceptable increase in procedural time. This facilitated the identification of additional cases of surgically curable unilateral PA, making ultra‐low dose ACTH a cost‐effective option to improve the diagnostic success of AVS. The results we report regarding ultra‐low dose ACTH AVS still should be interpreted with caution as they represent preliminary findings, requiring confirmation in larger, multicentre prospective studies with standardised long‐term follow‐up.

## Author Contributions

N.S. conceived and designed the study. C.A.P. and N.S. developed the analysis plan. E.X.Z.Y. and B.M. performed the AVS studies. S.G.F. performed adrenalectomies. C.A.P., M.P.S., M.M.D. and H.H. were involved in data management and C.A.P. in statistical analysis. C.A.P. drafted the manuscript. C.A.P., N.S. and R.J.M. edited the manuscript. All authors approved the final version of the manuscript.

## Ethics Statement

The project was prospectively approved by the St Vincent's Hospital Quality Assurance Sub‐committee of Human Research Ethics Committee (HREC) as a quality assurance study (QA 22031) and the same committee waived the need for patient informed consent.

## Conflicts of Interest

The authors declare no conflicts of interest.

## Supporting information


Table S1.

## Data Availability

The datasets generated during and/or analysed during the current study are available from the corresponding author on reasonable request.
