# Supplementary material for: Utility of Adrenal Vein Sampling With and Without Ultra‐Low Dose ACTH Infusion in the Diagnostic Evaluation of Primary Aldosteronism
Source: Endocrinol Diabetes Metab. 2024 Aug 29;7(5):e70001. doi: 10.1002/edm2.70001 (PMC11361314; doi:10.1002/edm2.70001)
Supplement: Supplementary file 1 — Table S1. [file EDM2-7-e70001-s001.pdf]

Supplementary Tables

Supplementary Table 1: Interfering medication washout protocol

Drug Class	Examples	Date to cease
Mineralocorticoid Receptor Antagonists	Eplerenone<br>Spironolactone	6 weeks prior to AVS
Loop Diuretics	Amiloride<br>Frusemide	4 weeks prior to AVS
Thiazide Diuretics	Hydrochlorothiazide<br>Indapamide	4 weeks prior to AVS
ACE Inhibitors	Captopril<br>Enalapril<br>Perindopril<br>Quinapril<br>Ramipril	2 weeks prior to AVS
Angiotensin Receptor Blockers	Candesartan<br>Losartan<br>Valsartan	2 weeks prior to AVS
Beta Blockers	Atenolol<br>Bisoprolol<br>Metoprolol<br>Sotalol	2 weeks prior to AVS
Calcium Channel Blockers	Amlodipine<br>Lercanidipine<br>Nifedipine	2 weeks prior to AVS

ACE, Angiotensin Converting Enzyme; K, potassium

Supplementary Table 2: AVS interpretation parameters, with and without ACTH stimulation

	Pre-ACTH	Post-ACTH
Selectivity Index (SI)	≥ 2.0	≥ 4.0
Lateralisation Index (LI)	≥ 3.0	≥ 4.0
Contralateral Suppression	≤ 1.0	≤ 1.0

Successful AV cannulation was defined by an AV to peripheral vein cortisol ratio ≥2.0 pre-ACTH and ≥4.0 post-ACTH (the selectivity index). Lateralisation was defined by an aldosterone to cortisol ratio of the dominant to non-dominant adrenal vein ≥3.0 pre-ACTH and ≥4.0 post-ACTH (the lateralisation index). Contralateral suppression was defined by an aldosterone to cortisol ratio of the non-dominant adrenal vein to the peripheral veins of ≤1.0

Supplementary Table 3: Aldosterone and cortisol levels of successful and failed AVS procedures

	Basal AVS				ACTH-stimulated AVS			
	LAV	LPV	RAV	RPV	LAV	LPV	RAV	RPV
Successful procedures, n=26								
Aldo (pmol/L)	5255 (1393,10605)	406 (346,798)	3910 (1165,13400)	392 (334,723)	81300 (19580,125450)	1155 (789,1523)	36240 (16590,112450)	1195 (732,1535)
Cortisol (nmol/L)	549 (377,1331)	140 (104,236)	566 (445,1109)	136 (89,195)	15005 (9575,22646)	488 (452,543)	21348 (15670,30164)	489 (428,533)
Failed procedures, n=11								
Aldo (pmol/L)	1170 (779,3275)	610 (424,769)	7260 (1483,13220)	613 (408,780)	9500 (3785,73600)	1470 (1160,1765)	61600 (2270,193200)	1430 (1111,1970)
Cortisol (nmol/L)	316 (281,385)	175 (153,248)	403 (202,427)	193 (115,207)	11791 (4662,14779)	444 (443,495)	17126 (1661,20022)	475 (443,507)

Values are median (interquartile range). AVS, adrenal vein sampling; LAV, left adrenal vein; LPV, left peripheral vein; RAV, right adrenal vein; RPV, right peripheral vein; Aldo, aldosterone.

Supplementary Table 4: AVS procedures with failed cannulation

	Basal AVS			ACTH-stimulated AVS			
Patient	SI (L)	SI (R)	LI	SI (L)	SI (R)	LI	Concordant
Pre failed, post unilateral (F/U), n=5							
1	1.83	6.47	2.01	6.66	47.36	6.25 (R)	No
2	1.91	25.74	19.32	40.15	60.19	42.85 (R)	Yes
3	1.92	1.95	10.31	31.07	37.92	47.53 (R)	Yes
4	1.45	1.98	48.65	20.71	37.39	78.48 (R)	Yes
5	1.64	2.09	26.68	9.24	36.11	10.59 (R)	Yes
Pre failed, post bilateral (F/B), n=2							
1	1.08	1.65	1.40	19.84	15.89	1.16	N/A
2	1.81	4.54	0.23	42.52	49.77	1.15	No
Pre failed, post failed (F/F), n=4							
1	2.69	1.08	23.35	23.17	1.08	11.82	N/A
2	1.85	1.15	27.38	25.25	0.95	13.78	N/A
3	2.84	1.17	2.64	9.66	1.60	1.60	N/A
4	1.79	3.45	11.16	1.96	7.40	2.12	N/A

AVS, adrenal vein sampling; SI, selectivity index; LI, lateralisation index; L, left; R, right.

Supplementary Table 5: Overall procedural time

	Basal and stimulated AVS	Stimulated AVS	*p*-value
Procedure duration (minutes)	143±33	98±36	<0.01

Values are mean ± standard deviation. Comparator (control) calculated from n=37 consecutive AVS procedures performed with ACTH only.

Supplementary Table 6: Procedural time by interventional radiologist

	Radiologist A	Radiologist B	*p*-value
Procedure duration (minutes)	141±32	150±34	0.47

Values are mean ± standard deviation.

Supplementary Table 7: Procedural time by experience

	Early cases	Later cases	*p*-value
Procedure duration (minutes)	147±39	142±31	0.70

Early cases, first 10 procedures; Later cases, last 27 procedures. Values are mean ± standard deviation.

Supplementary Table 8: Radiologists' cannulation success

	Pre-ACTH	With ACTH	*p*-value
Radiologist A	20/26 (77%)	24/26 (92%)	<0.01
Radiologist B	6/11 (55%)	9/11 (82%)	0.02

Pre-ACTH, basal AVS; with ACTH, stimulated AVS.

Supplementary Figures

Supplementary Figure 1: ACTH infusion protocol

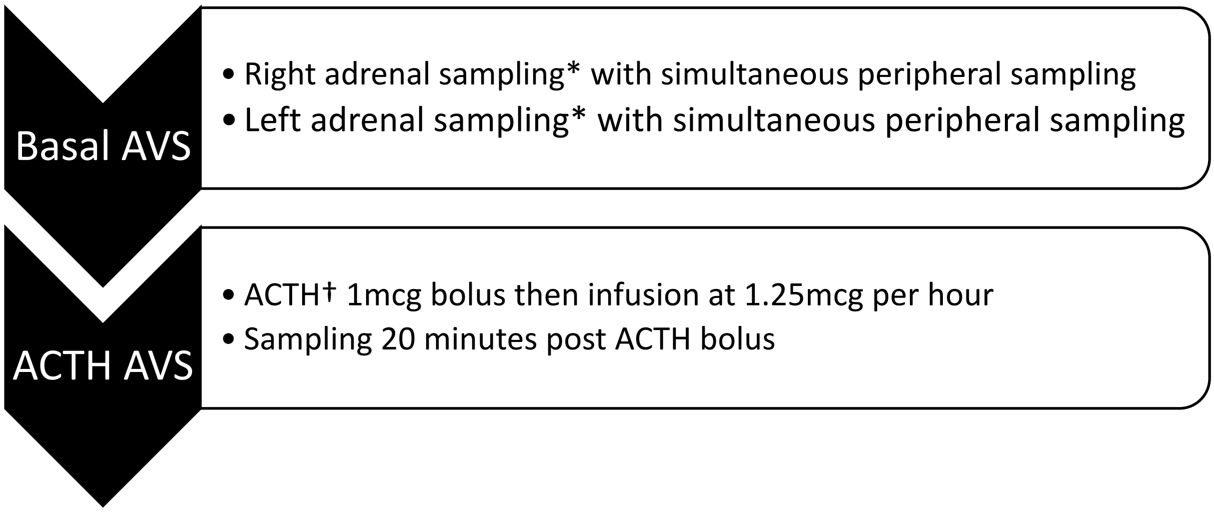

**Catheter placement confirmed utilising intra-procedural quick cortisol assay (QCA).*

†Add 250mcg Synacthen into 500mL 4% Gelofusine; Add 12.5mL of above solution into 500mL 0.9% normal saline; Bolus 80mL (1mcg) over 5 minutes, then continue at 100mL per hour (1.25mcg per hour) until end of procedure; Sampling commenced 20 minutes post ACTH bolus.
